# The role of LKB1 in prostate cancer: implications for tumor progression and therapy

**DOI:** 10.3389/fcell.2025.1629844

**Published:** 2025-08-12

**Authors:** Yuwei Liang, Hongliang Cao, Zhijun Tang, Shuxin Li, Gang Yang, Shuai Dong, Hao Du, Jinguo Wang

**Affiliations:** Department of Urology II, The First Hospital of Jilin University, Changchun, China

**Keywords:** LKB1, prostate cancer, signaling pathway regulation, epigenetic regulation, precision therapeutic targets

## Abstract

Liver kinase B1 (LKB1/STK11) is a key tumor suppressor that regulates cellular metabolism, epigenetic states, and multiple signaling pathways in prostate cancer (PCa). Recent studies reveal that both genetic and non-genetic LKB1 loss drives metabolic reprogramming, lineage plasticity, and treatment resistance, mainly through dysregulation of the AMP-activated protein kinase (AMPK)/mechanistic target of rapamycin (mTOR), signal transducer and activator of transcription 3 (STAT3), and Hedgehog (Hh) pathways. This review summarizes current evidence on LKB1-centered networks in PCa, highlighting the potential link between LKB1 inactivation, epigenetic remodeling, and aggressive tumor phenotypes. Special attention is given to recent studies on the impact of combined LKB1 and Phosphatase and Tensin Homolog (PTEN) loss on tumor differentiation. Finally, we discuss emerging therapeutic strategies aimed at the metabolic and epigenetic features of LKB1-deficient PCa, with a focus on the prospects for biomarker-driven precision medicine to address resistance and improve patient outcomes.

## 1 Introduction

Prostate cancer (PCa) is the second most commonly diagnosed malignancy in men and remains a leading cause of cancer-related deaths worldwide (Sung et al., 2021). While advances in local and systemic therapies, including surgery, radiotherapy, and androgen deprivation, have improved patient outcomes, a substantial proportion of patients with high-risk or advanced disease continue to face recurrence, metastasis, and progression to castration-resistant PCa (CRPC) (Moris et al., 2020; Kerkmeijer et al., 2021; Kishan et al., 2022). These clinical challenges highlight the ongoing need to clarify the molecular underpinnings of PCa and to identify novel, effective therapeutic targets.

Liver kinase B1 (LKB1, also known as STK11) has emerged as a master regulator of cell polarity, metabolism, and growth through its kinase activity and broad influence on downstream signaling pathways—including AMP-activated protein kinase (AMPK) and mechanistic target of rapamycin (mTOR) (Jansen et al., 2009; Shackelford and Shaw, 2009; Zeqiraj et al., 2009). While the tumor-suppressive function of LKB1 is well-established in several cancers, most notably lung adenocarcinoma and Peutz-Jeghers syndrome (Ji et al., 2007; Tacheci et al., 2021), the biology of LKB1 in PCa displays unique patterns (Kang et al., 2024). Unlike other tumors where LKB1 is frequently inactivated by direct genetic alteration, PCa is characterized by rare LKB1 mutations or deletions; instead, suppression more commonly results from epigenetic modulation and altered upstream signaling networks (Li et al., 2025).

In recent years, experimental studies have illuminated how loss of LKB1, particularly when coupled with key oncogenic events such as Phosphatase and Tensin Homolog (PTEN) deficiency, accelerates PCa progression, enhances metastatic potential, and influences lineage plasticity. These observations underscore the context-dependent and multifaceted roles of LKB1 within the prostate microenvironment (Hermanova et al., 2020; Li et al., 2025). This review synthesizes current knowledge regarding the regulation and function of LKB1 in PCa, contrasts its roles across different tumor types, and discusses the implications for prognostication and therapeutic intervention. We also highlight unresolved questions and future research directions that may help translate advances in LKB1 biology into improved clinical outcomes for patients with PCa.

## 2 Essential functional modules and cancer-related signaling networks of LKB1

LKB1 functions as a master regulator of cellular adaptation and integrity, exerting its effects—by forming a complex with the STE20-related kinase adaptor (STRAD) and MO25-alpha (CAB39)—through a broad network of serine/threonine kinases that enable cells to sense, integrate, and respond to environmental and metabolic stressors (Baas et al., 2003; Boudeau et al., 2003; Boudeau et al., 2004). Rather than operating in isolation, LKB1 orchestrates a dynamic signaling cascade, most notably by activating AMPK and a family of related kinases including microtubule affinity-regulating kinase (MARK), salt-inducible kinase (SIK), and NUAK family kinase (NUAK) (Kojima et al., 2007; Zagórska et al., 2010; Li et al., 2015). This extensive kinase network serves as both a cellular safeguard and a versatile signaling platform, crucially linking cellular stress sensing to adaptive responses. Upon metabolic or energetic perturbation leading to increased AMP:ATP ratio, LKB1 phosphorylates and activates the AMPK pathway, triggering a shift from energy-consuming biosynthetic programs to processes that restore adenosine triphosphate (ATP) and preserve cell viability (Shaw et al., 2004). This includes inhibition of mechanistic target of rapamycin complex 1 (mTORC1) signaling, induction of autophagy, and enhanced fatty acid oxidation, effectively allowing cells to endure nutrient scarcity and oxidative challenges (Hardie et al., 2006). Through these fundamental actions, LKB1 not only promotes metabolic homeostasis but also acts as a crucial brake on unchecked proliferative and anabolic signaling frequently observed in cancer.

Beyond metabolism, LKB1 plays a pivotal role in maintaining epithelial architecture, cellular polarity, and structural cohesion. By activating kinases such as MARK and regulating polarity complexes, it preserves apicobasal polarity and reinforces cell–cell junctions. The loss of LKB1 compromises these structures, facilitating cytoskeletal reorganization, detachment, and heightened cell motility—phenotypes intimately linked to tumor invasion and metastasis (Kojima et al., 2007; Goodwin et al., 2014; Mohseni et al., 2014). LKB1 also exerts sophisticated control over the cell cycle and cell survival via interactions with proteins like tumor protein p53 (p53), cyclin-dependent kinase inhibitor 1 (p21), and multiple cyclin-dependent kinases. Its regulatory web intersects with other key cancer pathways, including the Hippo- Yes-associated protein (YAP) and Wnt/β-catenin signaling axes, thereby influencing not only proliferation and differentiation, but also cell fate decisions such as senescence and apoptosis (Morton et al., 2010; Mohseni et al., 2014; Wang J. et al., 2015). Recent findings extend this regulatory reach to the tumor microenvironment, where LKB1 loss has been shown to modulate immune cell infiltration, inflammatory cytokine production, and stromal remodeling—all pivotal factors in tumor progression and therapeutic resistance (Gao et al., 2010; Koyama et al., 2016; Nguyen and Spranger, 2020). Loss or inactivation of LKB1 is a recurrent event in a variety of human cancers, such as lung adenocarcinoma, cervical carcinoma, and melanoma (Wingo et al., 2009; Liu et al., 2012; Mével-Aliset et al., 2025). Preclinical models have demonstrated that LKB1 deficiency synergizes with potent oncogenes, e.g., Kirsten Rat Sarcoma Viral Oncogene Homolog (KRAS) mutations, to promote rapid tumor growth, metabolic deregulation, and metastatic dissemination (Morton et al., 2010; Caiola et al., 2018). At the same time, the phenotypic consequences of LKB1 loss are shaped by cellular context, co-mutational landscape, tissue-specific metabolic dependencies, and microenvironmental factors. For example, genetic studies in mouse models have shown that the absence of LKB1 alone is insufficient to drive malignant transformation in many tissues, but dramatically enhances oncogenesis when combined with other mutations or environmental challenges (Ji et al., 2007).

**FIGURE 1:**
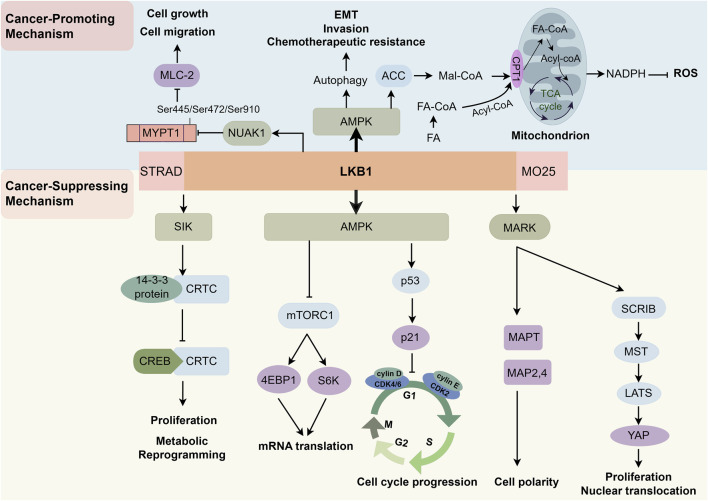
Schematic overview of the multidimensional regulatory network mediated by LKB1 in cancer. This diagram summarizes the dual roles of LKB1 as both a tumor suppressor and tumor promoter, with particular emphasis on mechanisms relevant to PCa. LKB1 exerts its kinase activity by forming a complex with STRAD and MO25. As a tumor suppressor, LKB1 activates AMPK to inhibit mTORC1-dependent biosynthesis, regulates p53 and p21 to enforce cell cycle arrest, maintains epithelial polarity and cytoskeletal integrity through the MARK pathway, interacts with the Hippo pathway to restrain YAP activity, and activates the LKB1/SIKs/CRTC axis to repress pro-tumorigenic transcription. Conversely, under certain conditions such as energy stress in PCa cells, LKB1/AMPK signaling enables metabolic reprogramming and redox balance to support tumor growth and survival, facilitates migration via NUAK1, and contributes to sphere formation and extracellular matrix remodeling, collectively promoting PCa metastasis and progression. Abbreviations: LKB1, liver kinase B1; AMPK, AMP-activated protein kinase; STRAD, STE20-related kinase adaptor; MO25, MOB1 Homolog; ACC, acetyl-CoA carboxylase; Mal-CoA, malonyl-coenzyme A; CPT1, carnitine palmitoyltransferase 1; FA, fatty acid; FA-CoA, fatty acyl-coenzyme A; TCA, tricarboxylic acid cycle; Acyl-CoA, acyl coenzyme A; NADPH, reduced nicotinamide adenine dinucleotide phosphate; ROS, reactive oxygen species; mTORC1, mechanistic target of rapamycin complex 1; NUAK1, NUAK family kinase 1; MYPT1, myosin phosphatase target subunit 1; MLC-2, myosin light chain-2; MARK, microtubule affinity regulating kinase; SIK, salt-inducible kinase; CRTC, CREB-regulated transcription coactivator; CREB, cAMP response element-binding protein; p53, tumor protein p53; p21, cyclin-dependent kinase inhibitor 1; CDK2/4/6, cyclin-dependent kinase 2/4/6; S6K, ribosomal S6 kinase 1; MAPT, microtubule-associated protein Tau; MAP2/4, microtubule-associated proteins 2/4; EMT, epithelial–mesenchymal transition; MST, mammalian Ste20-like kinase; LATS, large tumor suppressor kinase; YAP, yes-associated protein.

Altogether, the LKB1-controlled signaling network integrates metabolic, architectural, cell cycle, and environmental information to restrain oncogenic transformation and progression. It serves as a robust defense system ensuring that cells can properly adapt to stress while maintaining structural and proliferative order. However, as will be discussed in the following sections, the landscape of LKB1 signaling and its functional consequences can differ substantially across cancer types. In PCa, in particular, emerging evidence points to uniquely complex patterns of pathway regulation and biological outcome, warranting a more focused exploration. A schematic overview of the major molecular axes and cellular pathways governed by LKB1 is presented in Figure 1, highlighting the dual roles and the diverse cellular outcomes of its signaling. Pathways highlighted herein will be further discussed in the context of PCa in subsequent sections.

## 3 LKB1 in PCa

### 3.1 Dysregulation of LKB1 expression in PCa

The androgen receptor (AR) signaling pathway represents a cornerstone of PCa biology, orchestrating the proliferation and survival of luminal prostate cells and thereby driving tumor initiation and progression. This underpins the remarkable initial efficacy of androgen deprivation therapy (ADT) in controlling early-stage disease. However, tumor cells possess notable adaptive capacity. During extended ADT, they evade dependence on AR through lineage plasticity, culminating in CRPC (Lee et al., 2016; Bishop et al., 2017; Ku et al., 2017). Notably, CRPC is not a homogeneous entity. While neuroendocrine prostate cancer (NEPC) stands out as a classic example of lineage transformation, the majority of AR-negative tumors in clinical cohorts lack neuroendocrine features, instead being classified as double-negative prostate cancer (DNPC, lacking both AR and neuroendocrine markers) (Bluemn et al., 2017). These tumors are particularly aggressive; metastatic castration-resistant prostate cancer (mCRPC) patients with DNPC features treated with AR pathway inhibitors (ARPIs) often have strikingly poor survival (Lundberg et al., 2023). Yet, the molecular underpinnings driving DNPC’s biological aggressiveness remain poorly elucidated, presenting a formidable barrier to the development of effective precision therapies.

Unlike in several other solid tumors—such as lung adenocarcinoma—where LKB1 is frequently inactivated by homozygous deletion or mutation, such “classical” genetic events are rare in PCa. Instead, dysregulation of LKB1 in PCa appears to be orchestrated by epigenetic silencing, post-translational modifications, and crosstalk with other oncogenic alterations (Koseoglu et al., 2021). Large-scale profiling studies consistently report downregulation of LKB1 at both mRNA and protein levels in tumor tissue, with protein expression often reduced to less than half of that seen in benign prostate (Xu et al., 2014; Lu et al., 2015). Importantly, no recurrent coding sequence mutations were found even in deeply sequenced PCa cohorts, supporting a non-canonical model of LKB1 dysregulation (Koseoglu et al., 2021). These findings highlight that, unlike a straightforward “gatekeeper” tumor suppressor, LKB1’s role in PCa is dynamically shaped by the tumor milieu—including epigenetic modifications, protein stability, and crosstalk with other oncogenic alterations.

Notably, functional impairment of LKB1 (e.g., via epigenetic silencing) is significantly enriched in metastatic prostate cancer—implicating its role in advanced disease progression. Hermanova et al. pioneered the investigation of LKB1’s functional relevance in metastasis using an immunocompetent mouse model. Conditional deletion of LKB1 in prostate epithelial cells induced only mild prostate enlargement without tumorigenesis or survival deficits. Remarkably, LKB1 loss acted synergistically with PTEN loss to promote lineage plasticity and aggressive phenotypic transformation, including model-specific squamous differentiation in mice, and potently enhanced lung metastasis (Hermanova et al., 2020). This finding identifies LKB1 deficiency as a critical cooperative event driving metastatic progression and reveals its synergistic interaction with PTEN loss in regulating tumor lineage plasticity, thereby providing insights into DNPC pathogenesis.

Collectively, LKB1 dysregulation in PCa is primarily driven by non-genetic mechanisms—epigenetic silencing, post-translational modifications, and synergistic interactions with oncogenic pathways such as PTEN loss. This complexity underlies its context-dependent roles in PCa progression.

### 3.2 Crosstalk between LKB1 and key signaling pathways in PCa

**FIGURE 2:**
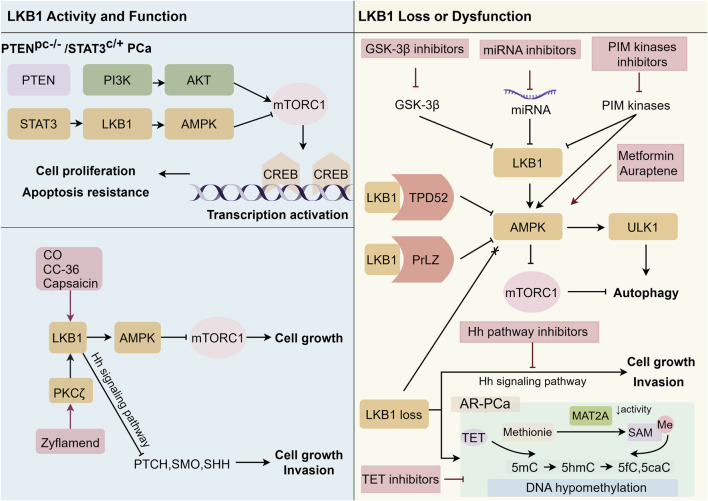
Therapeutic strategies for prostate cancer (PCa) are based on the regulatory mechanisms of Liver kinase B1 (LKB1) under different circumstances: In PCa cells with normal functionality, activation of the LKB1/AMP-activated protein kinase (AMPK) pathway strongly inhibits tumor proliferation. In Phosphatase and tensin homolog (PTEN)-deficient cells, persistent activation of Signal transducer and activator of transcription (STAT3) increases LKB1/pAMPK levels, thereby suppressing the mechanistic target of rapamycin complex 1 (mTORC1)/cAMP response element-binding protein (CREB) pathway. Additionally, eliminating inhibitors like Tumor Protein D52 (TPD52), Glycogen synthase kinase 3 beta (GSK-3β), MicroRNA (miRNA), PIM kinases, and Prostate Leucine Zipper (PrLZ)—along with suppressing DNA demethylation, activating AMPK, or inhibiting the Hedgehog (Hh) pathway—can strongly activate LKB1 or other tumor-suppressive pathways. This comprehensive strategy provides multi-level treatment options for PCa, offering new therapeutic opportunities in PCa management. Abbreviations: PCa: prostate cancer; PTEN: Phosphatase and tensin homolog; PI3K: Phosphoinositide 3-kinase; STAT3: Signal transducer and activator of transcription; LKB1: Liver kinase B1; AMPK: AMP-activated protein kinase; mTORC1: mechanistic target of rapamycin complex 1; CREB: cAMP response element-binding protein; CO: Carbon monoxide; CC-36: a unique anthraquinone derivative; PKCζ: Protein kinase C zeta; PTCH: Patched 1; SMO: Smoothened; SHH: Sonic hedgehog; GSK-3β: Glycogen synthase kinase 3 beta; miRNA: MicroRNA; TPD52: Tumor Protein D52; PrLZ: Prostate Leucine Zipper; ULK1: Unc-51 like autophagy activating kinase 1; MAT2A: Methionine Adenosyltransferase 2A; TET: Ten-eleven translocation; Me: Methyl group; SAM: S-adenosyl methionine; 5 mC: 5-methylcytosine; 5hmC: 5-hydroxymethylcytosine; 5 fC: 5-formylcytosine; Hh: Hedgehog; 5caC: 5-carboxylcytosine.

Beyond expression-level abnormalities, LKB1 exerts its oncologic influence through complex interactions with pivotal PCa signaling networks. Two of the most recurrent and significant events in prostate carcinogenesis are AR pathway alteration and PTEN inactivation (see Figure 2). AR and PTEN orchestrate cell growth and homeostasis; their disruption leads to uncontrolled proliferation and malignant transformation (Carver et al., 2011). Acting as an upstream activator of AMPK, LKB1 modulates energy sensing and cellular stress adaptation—but it also possesses AMPK-independent activities, including functional crosstalk with PTEN to reinforce tumor suppressive functions (Mehenni et al., 2005). Notably, recent studies reveal that Signal Transducer and Activator of Transcription 3 (STAT3), a key transcription factor and metabolic integrator, regulates LKB1 expression in advanced PCa. STAT3 associates with the LKB1 promoter to drive its transcription: STAT3 loss reduces LKB1/AMPK activity, compromising metabolic homeostasis, while its constitutive activation in specific contexts further enhances LKB1/AMPK signaling. These interactions form a regulatory axis centered on STAT3-LKB1-AMPK-mTORC1, a critical driver of resistance and aggressive progression in metastatic PCa. Modulating this axis, for example, via AMPK-activating agents like metformin in tumors with functional PTEN signaling, may offer new therapeutic avenues (Pencik et al., 2023).

Beyond these metabolic interactions, another salient pathway is Hedgehog (Hh) signaling, increasingly recognized for driving tumor stemness, metastatic potential, and therapeutic escape in PCa (Ruiz i Altaba, 2008; Peng and Joyner, 2015; Bushman, 2016). Preclinical studies show that LKB1 downregulation augments Hh signaling (e.g., through GLI1 activation), promoting tumorigenicity and invasion; reciprocally, robust LKB1 activity constrains GLI-mediated Hh signaling, helping suppress malignant phenotypes (Xu et al., 2014). The reciprocal regulation between these pathways not only reveals new aspects of PCa biology but also highlights novel therapeutic vulnerabilities.

### 3.3 LKB1: epigenetic regulation and therapy resistance in PCa

In recent years, significant progress has been made in molecular targeted therapy for prostate cancer, including poly (ADP-ribose) polymerase (PARP) inhibitors (such as olaparib and rucaparib) for mCRPC patients with homologous recombination repair (HRR) gene mutations, novel androgen receptor antagonists (apalutamide and darolutamide) used in mCRPC and metastatic castration-sensitive prostate cancer (mCSPC), as well as new technologies like prostate-specific membrane antigen (PSMA)-directed Proteolysis-Targeting Chimera (PROTAC) degraders and Lutetium-177 Prostate-Specific Membrane Antigen (Lu-PSMA) radioligand therapy. These advancements have significantly prolonged patients’ survival and improved the level of precision treatment (Hassan et al., 2021). Besides, inactivation of LKB1 exerts extensive epigenetic effects that remodel cancer cell properties and responses to therapy; additionally, its own expression is subject to epigenetic regulation, forming a regulatory loop (Borzi et al., 2020). As the most common epigenetic alteration in prostate cancer, DNA hypermethylation not only leads to the silencing of cell cycle regulatory genes but also results in promoter hypermethylation of genes involved in tumor invasion (Phé et al., 2010; Chin et al., 2011). Recent work further reveals that LKB1 inactivation is tightly linked to the loss of AR-dependence: single-cell transcriptomics show that AR-negative/low PCa displays marked signaling entropy, and LKB1 loss is accompanied by global DNA hypomethylation, predisposing cells to AR-independent states. Importantly, targeting the hypomethylated state—by inhibiting ten-eleven translocation (TET) enzymes or supplementing the key methyl donor S-adenosylmethionine (SAM)—can substantially suppress CRPC cell growth (Li et al., 2025). Systematic integration of metabolomic and epigenomic data catalogs DNA hypomethylation as a “druggable” vulnerability in DNPC, supporting the rationale for TET-targeted therapy in resistant disease (Nouruzi and Zoubeidi, 2025).

LKB1 is also a pivotal player in chemotherapy and radiotherapy resistance (Ou et al., 2012; He et al., 2017). Resistance to docetaxel, a first-line agent in mCRPC, is strongly associated with upregulation of the prostate leucine zipper (PrLZ) protein, which interacts with and functionally antagonizes LKB1/AMPK-mediated autophagy (Figure 2) (Tannock et al., 2004; Wang et al., 2004; Zhang et al., 2011). By impairing autophagy, PrLZ imparts survival advantage to PCa cells exposed to cytotoxic or endocrine stress. Targeting PrLZ, especially in combination with autophagy modulators, holds potential for overcoming docetaxel resistance (Zeng et al., 2018). Furthermore, KIF7, a Hedgehog pathway regulator, restrains PCa malignancy via the LKB1/PTEN/Protein Kinase B (AKT) axis. Reduced KIF7 expression diminishes LKB1/PTEN levels, hyperactivates AKT, and promotes paclitaxel resistance, whereas KIF7 restoration enhances chemosensitivity both *in vitro* and *in vivo* (Cheung et al., 2009; He et al., 2014; Wong et al., 2017).

Collectively, these findings underscore LKB1’s centrality in epigenetic reprogramming and chemoresistance, laying the groundwork for innovative combinatorial therapies. In summary, current evidence suggests that LKB1 integrates upstream environmental, metabolic, and genetic signals in the control of tumor cell fate, plasticity, metastatic potential, and therapy response in PCa. A deeper understanding of these LKB1-mediated networks may enhance our insights into lethal PCa progression and ultimately inform the design of new, context-specific treatment strategies.

## 4 LKB1-targeted therapeutic agents and strategies

In recent years, the LKB1 signaling axis has become an increasingly attractive target for novel PCa therapies. Both preclinical and translational studies reveal that precise modulation of this axis—by natural products, synthetic drugs, kinase network regulators, and non-coding RNAs—can reshape tumor metabolism and cell fate, offering new possibilities for precision treatment in PCa (Figure 2).

### 4.1 Natural products and metabolic modulators

Natural compounds are a rich source of molecules that modulate LKB1 and its downstream metabolic networks (Table 1). Capsaicin, the pungent compound in chili peppers, activates AMPK and suppresses proliferation in LKB1-positive PCa cells such as LNCaP and PC3. Intriguingly, in LKB1-deficient DU145 cells, the antiproliferative and pro-apoptotic effects of capsaicin are markedly reduced, especially at higher concentrations (80–160 μM for 24 h). Restoration of LKB1 expression in DU145 cells via lentiviral infection reinstates capsaicin’s effects, including induction of apoptosis and inhibition of AR signaling, highlighting its strict LKB1 dependence. Mechanistic studies show that capsaicin activates LKB1/AMPK signaling in a transient receptor potential vanilloid 1 (TRPV1)-dependent manner, suppressing mTOR activity and driving apoptosis. Furthermore, when combined with docetaxel, capsaicin enhances the suppression of PCa growth, suggesting a promising adjuvant role (Sánchez et al., 2022).

**TABLE 1:** Core mechanisms and efficacy comparison of three natural products in the treatment of PCa.

Comparison items	Capsaicin	Auraptene	Zyflamend
References	Sánchez, B.G. et al. (2022), Spain	Akasaka, Y. et al. (2023), Japan	MacDonald, A.F. et al. (2018), United States
Cell Lines	LNCaP, PC3 (LKB1-positive); DU145 (LKB1-null)	LNCaP, DU145 (LKB1-null), PC3, HEK-293	CWR22Rv1 (CRPC), HeLa (LKB1-null/CaMKK2+), HCT116
Treatment regimen	0–160 μM; 1 h (AMPK), 24 h (proliferation/apoptosis)	0–30 μM; 0–48 h (proliferation), 8 h (AMPK), 96 h (migration)	200 μg/mL; 30 min (AMPK), 24 h (inhibitor pretreatment)
Core mechanism	Activates AMPK via a TRPV1/LKB1-dependent, CaMKK2-independent axis	Activates LKB1-dependent AMPK by increasing the ADP/ATP ratio; suppresses mTOR-S6K pathway	Activates AMPK (LKB1-dependent); inhibits CaMKK2 through DAPK-mediated Ser^511^ phosphorylation
Upstream kinases/regulators	LKB1 (essential); TRPV1 required	LKB1 (essential); CaMKK2 not involved	LKB1 (essential); inhibits CaMKK2 (oncogenic driver)
Key efficacy indicators	80 μM induces 27% apoptosis in LNCaP; sensitivity restored in LKB1-overexpressing DU145	IC_50_ for LNCaP: 11.0 μM; 30 μM significantly inhibits migration (wound-healing)	∼40% ATP reduction; inhibits CaMKK2 and mTORC1 signaling
AR regulation	AR degradation (AMPK-independent)	Downregulates AR and PSA at mRNA and protein levels	Reduces AR nuclear localization (indirectly)
Experimental focus	Compares TRPV1+ vs. TRPV1– and LKB1+ vs. null cells	Evaluates AR-positive/negative and LKB1-dependent effects	Uses CRPC model (CWR22Rv1) with constitutive AR activity
Translational implications	Potential for combined radio/chemotherapy in TRPV1+ tumors	Promising for LKB1-positive PCa; low toxicity in preclinical models	Synergistic with metformin in reducing PSA in CRPC (clinical case data)

Abbreviations: AMPK, AMP-activated protein kinase; AR, androgen receptor; CaMKK2, calcium/calmodulin-dependent protein kinase 2; CRPC, castration-resistant prostate cancer; DAPK, death-associated protein kinase; HEK-293, human embryonic kidney 293 cells; HCT116, human colorectal carcinoma cell line 116; IC_50_, half maximal inhibitory concentration; LKB1, liver kinase B1 (also known as STK11); mTOR, mechanistic target of rapamycin; mTORC1, mechanistic target of rapamycin complex 1; PCa, prostate cancer; PSA, prostate-specific antigen; PTEN, phosphatase and tensin homolog; Ser^511^, serine at position 511; S6K, ribosomal protein S6 kinase; STAT3, signal transducer and activator of transcription 3; TRPV1, transient receptor potential vanilloid 1; PDX, patient-derived xenograft. Commonly used PCa, cell lines in this table include LNCaP, PC3, DU145, CWR22Rv1, and HeLa, each with specific genetic backgrounds relevant to mechanistic studies.

Other plant-derived agents, such as Zyflamend—a multi-component herbal preparation—have been shown to act on multiple nodes in the LKB1 pathway. In CRPC cell lines, Zyflamend enhances LKB1 activation (through increased PKCζ phosphorylation) and suppresses the oncogenic kinase CaMKK2 via a death-associated protein kinase (DAPK)-dependent mechanism. This dual regulatory action can be blocked by specific inhibitors, indicating mechanistic specificity and providing a conceptual framework for multi-pathway targeting (MacDonald et al., 2018). Auraptene, a natural coumarin derivative and potent AMPK activator, exerts antitumor activity by elevating the intracellular AMP/ATP ratio. Treatment with auraptene (3–30 μM, 8–24 h) downregulates mTOR-ribosomal S6 kinase (S6K) signaling, reduces lipid synthesis, and inhibits AR expression, collectively impeding PCa cell proliferation and survival (Akasaka et al., 2023). Additionally, metabolic byproducts such as carbon monoxide (CO), generated via the heme oxygenase-1 (HO-1) pathway, have demonstrated robust anticancer effects. CO upregulates LKB1, activates the AMPK pathway, suppresses mTOR activity, inhibits PCa proliferation and invasion, and induces apoptosis *in vitro*. *In vivo* experiments confirm these effects, with tumor growth suppression significantly attenuated in LKB1 knockdown models, emphasizing LKB1’s centrality in the CO-mediated antitumor response (Loboda et al., 2015; Yan et al., 2018). Taken together, these examples highlight how diverse natural and metabolic agents converge on the LKB1 axis, revealing metabolic vulnerabilities that can be leveraged for future precision therapies.

### 4.2 Synthetic drugs and combination therapy strategies

Metformin, a first-line treatment for type 2 diabetes mellitus, has attracted attention for prostate cancer (PCa) therapy. Its direct anticancer mechanisms include LKB1/AMPK activation, mTOR activity inhibition, protein synthesis suppression, p53/p21-mediated apoptosis and autophagy induction, and reduced blood insulin levels (Ahn et al., 2020). Studies show that in androgen receptor-positive cell lines, metformin downregulates androgen receptor (AR) protein expression dose-dependently and inhibits AR signaling by reducing AR mRNA levels. This supports metformin’s role as a potential adjunct to androgen deprivation therapy (Demir et al., 2014; Wang Y. et al., 2015). However, a recent randomized phase 3 trial demonstrated that metformin did not reduce metabolic syndrome (MS) risk in PCa patients receiving ADT, though it may mitigate ADT-related complications (Eigl et al., 2025).

In the field of natural product development, anthraquinone-based compounds have garnered significant interest due to their broad biological activities, including antitumor effects (Akkol et al., 2021; Marrero et al., 2022; Tikhomirov et al., 2022). Derivatives like CC-36, a disubstituted anthraquinone, directly activate the LKB1/AMPK pathway, inhibit mTOR signaling, and induce G1-phase arrest and apoptosis in hormone-refractory metastatic PCa (HRMPC) cells. When combined with the AKT inhibitor MK2206, CC-36 synergistically blocks AKT and LKB1/AMPK/mTOR pathways, enhancing cell death and providing a rational polypharmacologic strategy to overcome resistance (Hsu et al., 2014).

Combination approaches targeting metabolic and AR signaling are increasingly studied. Metformin combined with enzalutamide reduces proliferation and viability in enzalutamide-resistant CRPC lines. Transcriptomic analysis suggests this involves Rat sarcoma viral oncogene homolog (Ras)-MAPK pathway downregulation and mitochondrial function impairment, though *in vivo* tumor growth suppression was inconsistent. These findings highlight the value—and translational challenge—of integrating metabolic regulators into combination regimens (Simpson et al., 2025).

### 4.3 Kinase interactions and gene-targeted interventions

Within the LKB1-associated signaling network, multiple key molecules display distinct interaction mechanisms. Studies have shown that the oncoprotein tumor protein D52 (TPD52), which is amplified at the chromosomal locus 8q21, undergoes aberrant overexpression in advanced PCa and drives tumor growth and cell proliferation through nuclear factor kappa-B (NF-κB) signaling (Dasari et al., 2017). Mechanistic studies demonstrate that Tumor Protein D52 Isoform 3 (TPD52L3)interacts with STRADα, a critical component of the LKB1 complex, via its 170–184 extended region, while its specific residues directly bind LKB1’s autophosphorylation sites Ser^325^/Thr^336^ (S325/T336). This dual interaction inhibits LKB1 kinase activity, leading to AMPK inactivation and downstream signaling dysregulation. Targeting this mechanism through combined AMPK activators or novel therapies disrupting TPD52/LKB1 interactions may provide therapeutic breakthroughs for TPD52-overexpressing tumors (Khilar et al., 2023).

The PIM family of kinases (PIM1/2/3) also modulates LKB1 via phosphorylation at serine 334 (Ser^334^), influencing AMPK activation and tumor proliferation. Complete inhibition or genetic deletion of PIM kinases significantly upregulates AMPK activity. Interestingly, dual knockout of LKB1 and PIM kinases exerts a synergistic tumor-suppressive effect, suggesting that indirect targeting of the LKB1/AMPK axis via PIM inhibition could benefit selected patients (Mung et al., 2021). The regulatory mechanism of glycogen synthase kinase 3β (GSK-3β) exhibits multi-layered characteristics. This kinase dynamically regulates AMPK signaling by restricting LKB1 nucleocytoplasmic transport and LKB1/STRAD/MO25 complex formation. GSK-3β inhibition lowers the ATP/ADP ratio by modulating energy metabolism, thereby triggering LKB1-dependent AMPK activation and subsequent inactivation of the mTOR pathway (Sun et al., 2016). Notably, under serum deprivation, GSK-3β′s regulation of LKB1 predominates, exerting stronger effects than calmodulin-dependent protein kinase β (CaMKKβ) signaling (Kaidanovich-Beilin and Woodgett, 2011; Beurel et al., 2015; Sun et al., 2016). Based on this mechanism, combining GSK-3β inhibitors—such as Tideglusib—with vascular disrupting agents (VDAs) enhances synergistic therapeutic effects through amplified tumor energy stress and vascular targeting, presenting an innovative combinatorial strategy for clinical translation (Mita et al., 2013; Porcù et al., 2014).

### 4.4 Non-coding RNA regulation and emerging targets

Non-coding RNAs, particularly microRNAs (miRNAs), represent an emerging and important regulatory layer over the LKB1 signaling circuit. MicroRNA-744 (miR-744) binds directly to the 3′untranslated region (3′UTR) of LKB1 mRNA, downregulating its expression. This reduces AMPK activation, induces aberrant mTOR signaling, accelerates cell cycle progression, and promotes PCa proliferation. Targeted inhibition of miR-744 could restore LKB1-mediated tumor suppression and provide a basis for novel miRNA-interference therapies (Zhang et al., 2019).

More broadly, miRNAs modulate AR signaling and significantly impact ADT resistance in PCa. Exploiting miRNAs in combination regimens offers dual benefits—overcoming castration resistance and serving as prognostic biomarkers for therapy stratification. Profiling miRNA expression in CRPC patients before treatment may improve ADT precision and identify patients at risk for rapid progression (Ebrahimi et al., 2021).

## 5 Future directions: translational potential of LKB1 in PCa

The translational landscape of LKB1 in PCa is rapidly expanding, presenting new opportunities for personalized medicine and overcoming therapy resistance. Recent advances in single-cell transcriptomics and spatial proteomics have begun to illuminate the cellular heterogeneity and lineage plasticity induced by LKB1 inactivation. Integrating such multi-omics technologies with functional studies of LKB1-associated pathways—particularly the interplay between the STAT3/LKB1/mTORC1/CREB axis and canonical PCa drivers—will be instrumental in uncovering actionable therapeutic targets (Pencik et al., 2023). At the same time, the growing appreciation of the role of epigenetic remodeling and metabolic reprogramming in LKB1-deficient contexts opens the door to novel interventions, including the use of DNA methylation inhibitors, TET enzyme blockade, AMPK activators, and the strategic supplementation of key metabolites like SAM (Nouruzi and Zoubeidi, 2025).

Practical translation, however, requires a nuanced understanding of the phenotypic consequences of LKB1 loss. Many LKB1-deficient tumors exhibit not only profound changes in autophagy and metabolism, but also immune escape and multidrug resistance. This complexity highlights the promise of rational combination regimens that pair metabolic and epigenetic modulators—such as Methionine Adenosyltransferase 2A (MAT2A) or Histone Deacetylase 6 (HDAC6) inhibitors or metformin—with immunotherapies, exploiting synthetic lethal interactions or targeting secondary vulnerabilities. For example, strategies that integrate Monopolar Spindle 1 Kinase (MPS1) inhibition to activate the Stimulator of Interferon Genes (STING) pathway, or combine TET inhibition with SAM supplementation, are emerging as potential means to restore immune surveillance and reverse lineage plasticity. These approaches underscore the importance of robust preclinical validation, ideally leveraging advanced models such as patient-derived xenografts and bone metastasis systems that mirror the clinical realities of metastatic PCa. At the core of these efforts lies the intricate crosstalk between metabolism and epigenetic regulation under LKB1 deficiency. Alterations in enzymes and cofactors—including MAT2A, SAM, and α-ketoglutarate—not only affect DNA and histone methylation but also shape the tumor’s adaptive landscape in the face of therapy. Deeper mechanistic insights into how these metabolic-epigenetic circuits contribute to subtype transitions and resistance could reveal entirely new therapeutic windows, potentially allowing resensitization to antiandrogen therapies through tailored metabolic interventions.

As this field moves forward, the identification and clinical validation of robust biomarkers—ranging from STRADβ and p-CREB protein levels to DNA methylation signatures and LKB1-driven transcriptomic classifiers—will be crucial for risk stratification and patient selection. Large-scale, well-annotated clinical datasets remain a pressing need to support these advances and enable biomarker-driven precision therapy. Importantly, translating these laboratory discoveries into the clinic will require creative approaches to circumvent multidrug resistance, which often accompanies LKB1 inactivation. Combination strategies—such as using metformin alongside next-generation AR pathway inhibitors and immune checkpoint blockade, or learning from therapeutic successes in other tumor types (e.g., applying sotorasib in LKB1-deficient settings)—offer tangible hope, particularly for patients with aggressive or refractory disease marked by high levels of STAT3 or CREB activity. Ultimately, bridging the gap between mechanism and clinic may benefit from a multidimensional strategy that links molecular, metabolic, and immune features of PCa. Continued exploration of metabolism–epigenetics–immunity crosstalk around LKB1 could help further refine therapeutic approaches for metastatic and treatment-resistant PCa (Garje et al., 2025).

## 6 Conclusion

This review provides an integrated and nuanced overview of the pivotal role of LKB1 in PCa, highlighting its multifaceted functions at the intersection of tumor metabolism, epigenetic regulation, and signal transduction. We summarize evidence demonstrating that LKB1 deficiency not only drives oncogenesis and therapeutic resistance—through AMPK/mTOR axis disruption, epigenetic dysregulation such as DNA hypomethylation, and maladaptive crosstalk with key pathways like STAT3 and Hedgehog—but also underpins dramatic phenotypic consequences, including lineage plasticity and squamous differentiation, particularly when combined with PTEN loss. By synthesizing advances from molecular mechanistic studies to preclinical and translational research, this review charts a conceptual path from basic biological understanding toward innovative clinical intervention. We emphasize the promise of targeting the metabolic–epigenetic axis using rationally designed pharmacological agents (both natural and synthetic), novel gene-based interventions, and combination regimens, particularly in the context of PCa subtypes defined by LKB1 status and associated molecular alterations. Looking ahead, integrating single-cell and multi-omics technologies, developing robust biomarkers for patient stratification, and rigorously validating therapeutic strategies in clinically relevant models will be critical for translating these insights into precision medicine. Ultimately, continued elucidation of LKB1-centered networks may help overcome resistance, inhibit disease progression, and ultimately improve outcomes for patients with advanced PCa.
